# Sharing Medical Big Data While Preserving Patient Confidentiality in Innovative Medicines Initiative: A Summary and Case Report from BigData@Heart

**DOI:** 10.1089/big.2022.0178

**Published:** 2023-12-14

**Authors:** Megan Schröder, Sam H.A. Muller, Eleni Vradi, Johanna Mielke, Yvonne M.F. Lim, Fabrice Couvelard, Menno Mostert, Stefan Koudstaal, Marinus J.C. Eijkemans, Christoph Gerlinger

**Affiliations:** ^1^ The Institute for Medical Information Processing, Biometry, and Epidemiology (IBE), Ludwig-Maximilians-Universität München, Münich, Germany.; ^2^ Julius Center for Health Sciences and Primary Care, University Medical Center Utrecht, Utrecht University, Utrecht, The Netherlands.; ^3^ Biomedical Data Science II, Bayer AG, Berlin, Germany.; ^4^ Research and Early Development, Bayer AG, Wuppertal, Germany.; ^5^ Institute for Clinical Research, National Institutes of Health, Selangor, Malaysia.; ^6^ Institut de Recherches Internationales SERVIER (I.R.I.S.), Suresnes, France.; ^7^ Division of Heart and Lungs, Department of Cardiology, University Medical Center Utrecht, Utrecht University, Utrecht, The Netherlands.; ^8^ Department of Cardiology, Groene Hart Ziekenhuis, Gouda, The Netherlands.; ^9^ Clinical Statistics and Data Insights, Bayer AG, Berlin, Germany.; ^10^ Department of Gynecology, Obstetrics and Reproductive Medicine, University Medical School of Saarland, Homburg/Saar, Germany.

**Keywords:** sharing individual patient data, summary statistics, health data research collaborations, patient data privacy, data transparency, responsible data governance

## Abstract

Sharing individual patient data (IPD) is a simple concept but complex to achieve due to data privacy and data security concerns, underdeveloped guidelines, and legal barriers. Sharing IPD is additionally difficult in big data-driven collaborations such as Bigdata@Heart in the Innovative Medicines Initiative, due to competing interests between diverse consortium members. One project within BigData@Heart, case study 1, needed to pool data from seven heterogeneous data sets: five randomized controlled trials from three different industry partners, and two disease registries. Sharing IPD was not considered feasible due to legal requirements and the sensitive medical nature of these data. In addition, harmonizing the data sets for a federated data analysis was difficult due to capacity constraints and the heterogeneity of the data sets. An alternative option was to share summary statistics through contingency tables. Here it is demonstrated that this method along with anonymization methods to ensure patient anonymity had minimal loss of information. Although sharing IPD should continue to be encouraged and strived for, our approach achieved a good balance between data transparency while protecting patient privacy. It also allowed a successful collaboration between industry and academia.

## Introduction

The Innovative Medicines Initiative (IMI) is the world's largest public–private partnership in the life sciences with the goal of improving patient health through collaborative research.^1–3^ Industry, academia, patients, health care providers, and several other partners have come together to reach this goal. BigData@Heart is one disease-specific project within IMI and falls under the umbrella program, Big Data for Better Outcomes (BD4BO).^4,5^ BD4BO is data centered, bringing together data sets and maximizing their use with collaborative research to answer disease-specific questions. In BigData@Heart, the focus is heart failure, acute coronary syndrome, and atrial fibrillation.

There are several methods that can be used to pool data including sharing individual patient data (IPD) or using a federated data analysis; however, these methods were not feasible in BigData@Heart due to data privacy concerns and lack of a common data format for clinical trials to allow federated data analysis. The next best option was to pool summary statistics from five heart failure clinical trials, provided by three different industry partners. Each institution kept the data on its premises and summary statistics were shared through contingency tables, a type of frequency distribution table. The main limitation to not sharing individual data is the information loss that may occur.^6,7^

The potential loss of information from sharing summary statistics through contingency tables was tested by comparing empirical cumulative distribution functions (ECDFs). The structure of this article is as follows. First, we explain the challenges to vested approaches for sharing IPD in research consortiums like BigData@Heart. Second, we outline the approach of sharing summary statistics that was tested as an alternative to established data sharing methods. Third, we present the results of potential loss of information from this approach. Fourth, we discuss in what ways sharing summary statistics could be a helpful alternative given practical challenges encountered in sharing data in large-scale health data research consortiums.

## Background

Sharing individual-level data, especially data from clinical trials, is well recognized as the future in medical research. Considering their role in determining evidence-based medicine and public health policies, clinical trial data sharing is particularly important.^8–15^ There are numerous benefits to sharing clinical data, including reducing duplicate trials, increasing transparency and validity,^16–18^ higher generalizability of results,^19^ and more efficient usage of secondary data.^10,11,13,16^ However, disadvantages of sharing data are the difficulties in ensuring data privacy, poor compensation to the data generators, and the risk of data mishandling and misinterpretation.^11,20–23^

Patients' medical data collected in registries or clinical trials are personal data that require special protection under privacy and data protection regulations.^24^ In the EU, sharing such sensitive personal data is prohibited by the General Data Protection Regulation (GDPR) (EU 2016/679), except from where exemptions laid down in national law allow for the processing for scientific research purposes.^25^ Several solutions have been proposed such as new web-based networking technologies, database management systems, and review committees for deidentification.^6,16,21,26,27^

Novel techniques using deep learning and blockchain techniques are being proposed for privacy preservation of centralized servers; however, the current conventional central medicinal information storing systems are still considered inadequate.^28^ Although the benefits generally outweigh the risks in most cases, the feasibility of sharing data is still very low due to underdeveloped guidelines, lagging harmonization of principles and norms, and lack of political support.^8,11,12,14,17,20,29–32^

Several guideline frameworks have been presented, such as the WMA Declaration of Taipei (2016), the CIOMS International Ethical Guidelines for Health-Related Research Involving Humans (2016), and the GA4GH Framework for Responsible Sharing of Genomic and Health-Related Data (2014).^22,23,33,34^ However, they have been criticized for being abstract principles open to interpretation.^22,23^ In addition, the services available to anonymize or deidentify IPD are still quite expensive and time consuming, as is the review process to gain data access.^20,32^

Another option to share data for research is a federated approach that keeps the data onsite with the data owners. Federated analytics (FA) is emerging as a new paradigm to address the data governance and privacy issues related to medical data sharing. FA allows statistical analysis including machine-learning models without exchanging the underlying data sets. There are still unresolved issues in FA as sharing the aggregated data can leak sensitive personal information that allows reidentification, membership inference, and feature reconstruction.

Several solutions have been proposed using techniques of differential privacy (diffP), secure multiparty computation, and homomorphic encryption; however, there are still limitations involved.^28,35^ To perform a federated data analysis, each data set must be harmonized to a common data. To harmonize the data, the Observational Medical Outcomes Partnership format was proposed as the common data model to be used in BigData@Heart.^36^ This format was created by Observational Health Data Sciences and Informatics (OHDSI),^37^ a similar multistakeholder interdisciplinary health data research collaboration based in Columbia University.

The common data model allows heterogeneous data sets to be converted into one common data model. There are several benefits to using a common data model including increased sample sizes to improve robustness and generalizability of results,^18,19^ ease of analysis,^30,38^ more efficient use of existing data,^11,16^ and facilitation of collaboration between unlikely partners.^16,29,39^

However, initially transforming a data set is time consuming and demands strong collaboration between partners to understand the ability of the data set to be transformed.^16,29,30^ The drawbacks of federated analysis include a loss of information in transforming a data set and that a federated data system needs to be implemented along with secure servers at each data site. A federated data system is a type of meta-database management system that allows remote immediate retrieval of statistical analyses.^16,27,40^ There are several benefits to this, including improving data privacy by not sharing IPD, and allowing investigators to remotely analyze data in real time at their own convenience.^16^

This approach allows data privacy to be maintained while keeping large sample sizes and robust results. FA still requires processing of sensitive personal data by the controller. The controller could be allowed to do this based on Article 5(1)(b) GDPR, which allows for an exception to the principle of purpose limitation, or another legal basis in the GDPR or national law. Lastly, there is no common data model for clinical data, although OHDSI is currently developing one in collaboration with The Hyve, a Netherlands-based data infrastructure and informatics service provider. For these reasons, BigData@Heart sought another solution to pool data from heterogeneous clinical trial data sets.

## Materials and Methods

The analysis strategy in case study 1 was to pool the data from five different heart failure with reduced ejection fraction (HFrEF) randomized controlled trials (RCTs) to have one representative population of HfrEF patients enrolled in RCTs. BEAUTIFUL and SHIFT were phase III ivabradine trials (*n* = 15,732),^6,27^ FAIR-HF and CONFIRM were phase III and phase IV studies on intravenous iron supplementation (*n* = 763),^29,30^ and PANTHEON was a phase II trial for neladenosone bialanate (*n* = 427).^41^ For each of the trials, ethics approval and written informed consent were obtained by the respective investigation.

To pool baseline characteristics without sharing IPD, the first step was to harmonize the variables by identifying shared variables between data sets and naming a standard unit. A standard excel template was shared with each data owner detailing the units and variables. Each variable was recorded and shared as continuous, categorical, or both. Continuous variables are presented as mean and standard deviation, and categorical values are presented in frequencies and percentages. When a variable was collected categorically, it was collected through a contingency table that leads to cases of small cell counts for the smaller trials.

To maintain patient anonymity, each cell with a count of 0, 1, 2, or 3 was automatically censored to 2. The summary statistics from each data set were then considered anonymized if they were an aggregate of at least three data points.^42^ Then, the anonymized counts from each of the five RCTs were shared to one central location where the data were then combined to form one RCT population. This anonymization was used in a previous publication to compare characteristics between the HFrEF RCT patients and patients found in two heart failure registries.^43^ Mean and proportion differences between each group were calculated and reported as significant based on their corresponding 99% confidence intervals (CIs).

To test the potential of minimal loss of information with this sharing and anonymization method, two methods were used in this study to statistically compare the original variables with the anonymized variables. First, a chi-square test was used to compare the distribution of the original variables in the combined RCT population with the distribution of the respective anonymized variable. A nonsignificant *p*-value would indicate that the loss of information was minimal. Second, the ECDF was compared between an anonymized and original variable in a single data set to more granularly test whether anonymization affected the distribution of the variable.

The corresponding CI of the original variable ECDF was calculated to test whether the ECDF of the anonymized variable was statistically different than the ECDF of the original variable. The PANTHEON data set was selected for the ECDF analysis as it was one of the smaller data sets used in the analysis that, therefore, had several cell counts needing anonymization. The following variables were selected based on low cell counts and whether they were used in the previous analysis: age, creatinine, and hemoglobin. The original cuts used for each variable are recorded in Table 1.

**Table 1.** Variables with low cell counts needing anonymization and associated chi-square test between original and anonymized variable for the five randomized controlled trials

Variable	Interval cut	Chi-squared test^a^
Age (years)	18–25, 26–30, 31–35, 36–40, 41–45, 46–50, 51–55, 56–60, 61–65, 66–70, 71–75, 76–80, 81–85, 86–90, 91–95, 96+	0.9262
Creatinine (μmol/L)	<90, 90–109, 110–129, 130–149, 150–169, 170–209, 210–249, ≥250	0.9614
Hemoglobin (g/dL)	≤8.0, 8.0–10.9, 11.0–12.9, 13.0–14.9, 15.0–16.9, ≥17.0	0.9798

^a^ Chi-square test was calculated between the original population and the anonymized population.

For the ECDF analysis, even smaller cuts of the variables were tested to assess the level of granularity of information that could have been shared. For age, loss of information was assessed when the variable was cut at 1- and 3-year intervals. For hemoglobin, intervals of 0.5 and 1.0 g/dL were tested. For creatinine, intervals of 5 and 10 μmol/L were tested. If the anonymized variable's ECDF stayed within the CI bands of the original variable's ECDF, then loss of information was considered minimal. Statistical analysis was performed using the R statistical software version 3.6.1.^44^

## Results

**FIG. 1.**
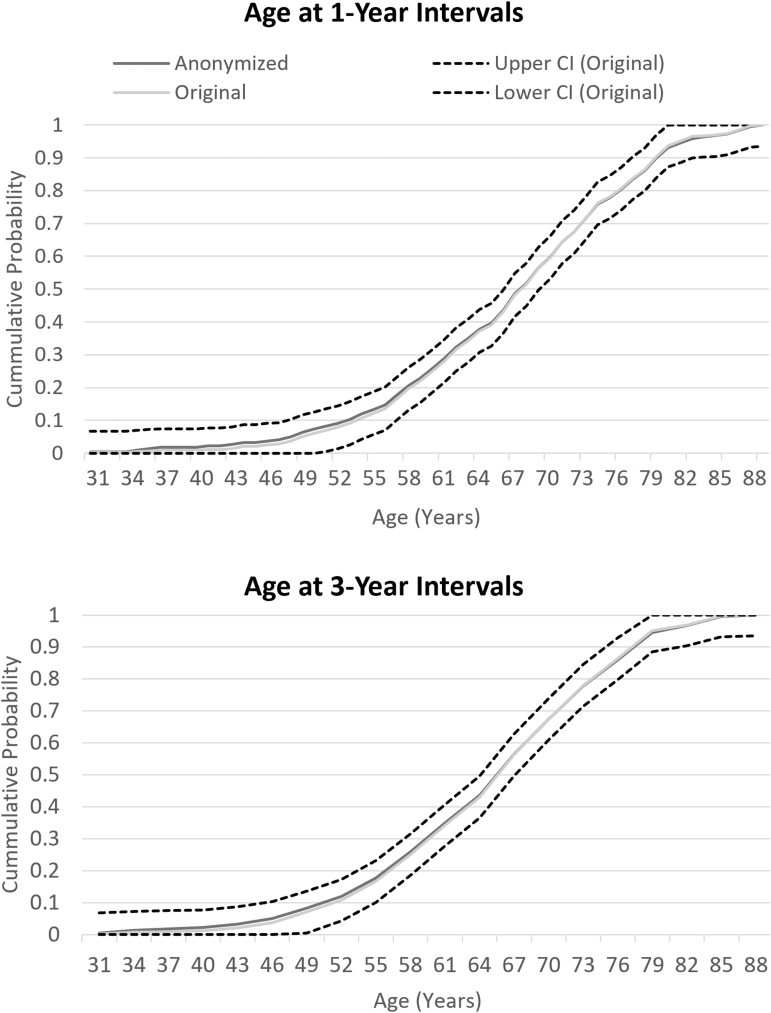
ECDF of age cut at 1- and 3-year intervals. The original and anonymized variable are compared with the CI of the original variable. The anonymized variable's ECDF stays within the CI bands of the original variable showing minimal loss of information with anonymization. CI, confidence interval; ECDF, empirical cumulative distribution functions.

**FIG. 2.**
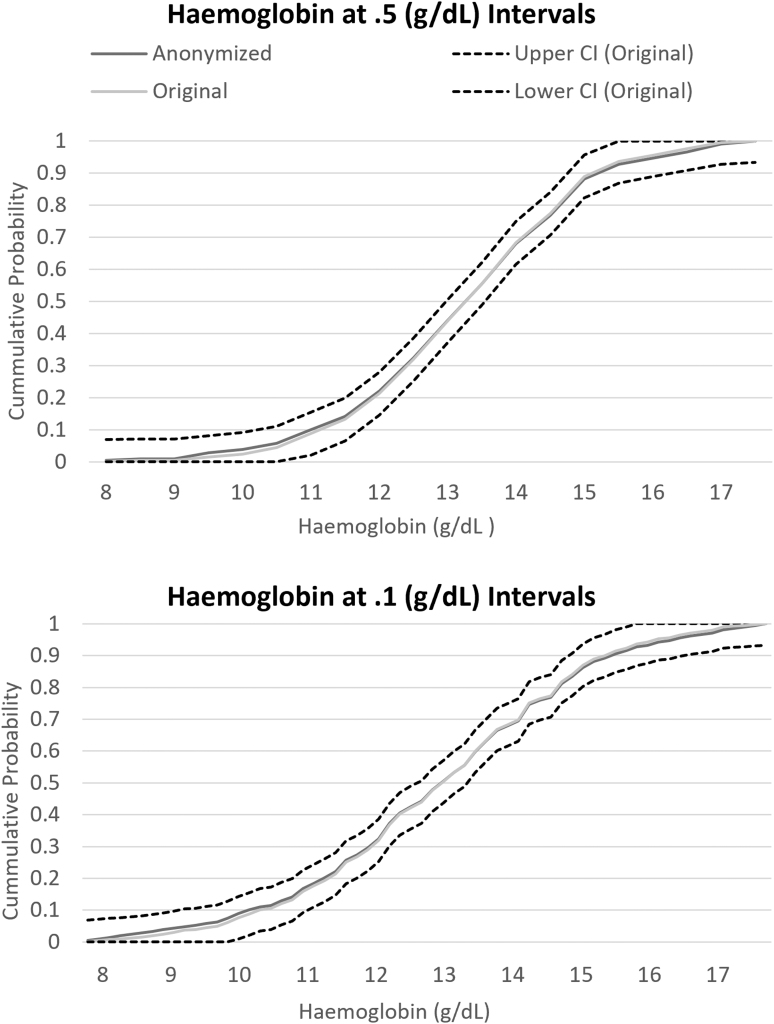
ECDF of hemoglobin cut at 0.5 and 0.1 g/dL intervals. The ECDF of the original and anonymized variable is compared with the CI of the original variable's ECDF. The anonymized variable's ECDF stays within the CI bands of the original variable showing minimal loss of information with anonymization.

**FIG. 3.**
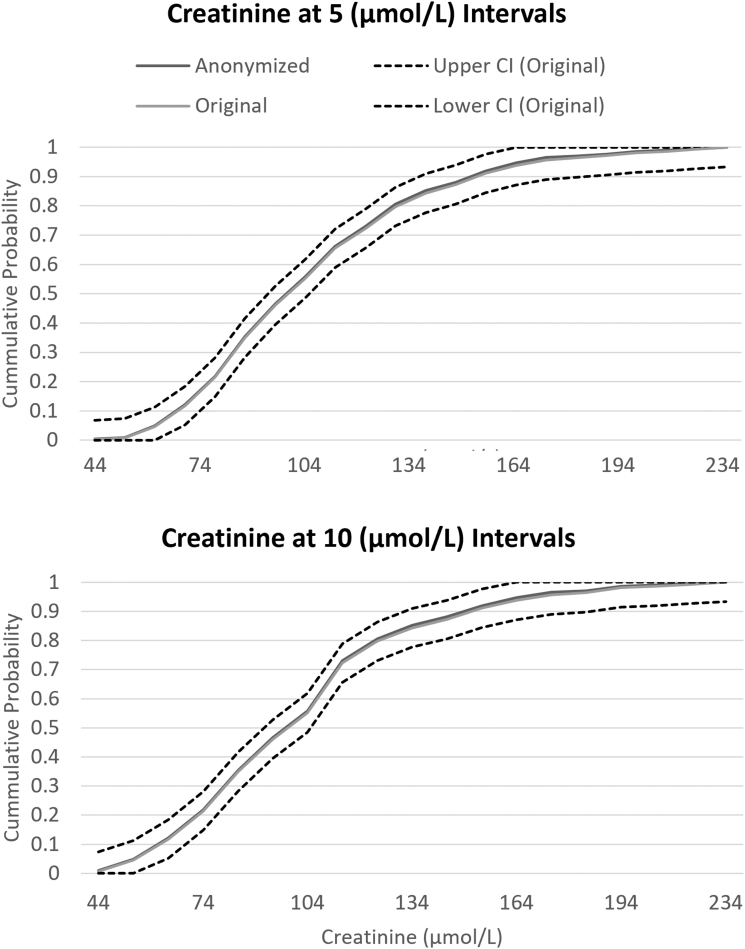
ECDF of creatinine cut at 5 and 10 μmol/L intervals. The ECDF of the original and anonymized variable is compared with the CI of the original variable's ECDF. The anonymized variable's ECDF stays within the CI bands of the original variable showing minimal loss of information with anonymization.

The chi-square test comparing the distribution between the anonymized and original variable was insignificant for age (*p* = 0.9262), creatinine (*p* = 0.9614), and hemoglobin (*p* = 0.9798) in the total combined RCT population (Table 1). In each variable and for every interval tested in the PANTHEON data set, the ECDF of the anonymized variable stayed within the CI bands of the original variable. At the smallest intervals where the variables experienced several cases of low cell counts, the anonymized variable maintained minimal loss of information (Figs. 1–3).

## Discussion

Our approach in pooling the aggregated summary statistics from each data set was a valid approach, resulting in minimal loss of information. It is important to note that there is not a “one-size fits all” approach in data sharing, especially in a project with diverse clinical trials from industry.^17^ Although the culture is changing, sharing data directly between at times competing industry partners and academic partners is hardly routine and still has numerous challenges with data privacy and a lack of resources.^8,11,17,21,23,26,29,31^

The problems experienced in sharing IPD could have been mitigated if in the planning phase of a clinical trial the principles that govern how data may be shared with other projects had been elaborated and agreed on. This coincides with the common proliferation of a general lack of attention and awareness for the importance of harmonizing underlying principles and norms that facilitate the sharing of data sets. Although this is often perceived as marginal, it undergirds the smooth interaction between research partners that is needed for cooperation in data sharing. Efforts to establish responsible and sustainable data sharing governance should meaningfully involve pertinent stakeholders, including patients.

Their active integration in different stages of data governance facilitates the establishment of common grounds necessary for the required cooperation.^45^ As suggested for instance by the GA4GH,^46^ bridging differences and perspectives between divergent interests of consortium partners and between researchers and stakeholders could take place in the data access review process in data access committees.

Although our approach of sharing summary statistics through contingency tables was a valid approach, it was chosen out of feasibility and lack of other options. Therefore, this approach highlights that greater effort on harmonization of principles and norms and interoperability forms a practical necessity to further smooth sharing and linkage of data sets. There are several strengths and limitations to this approach. The main limitation is the, although minimal, information loss. This study proved that there is minimal loss of information, and that data could have been shared even more granularly while maintaining patient anonymity.

This approach was also the easiest option in terms of initial planning and execution of the analysis. Fully harmonizing the data would have required substantial effort from the entire consortium to transform each data set into an agreed-upon format.^16,17,29^ Using a federated analysis would have additionally required adequate servers and programs to run the analysis on any data set in any location. Although initially daunting, these tasks to allow a federated data analysis would have cumulatively saved effort and time, especially when multiple analyses are planned. Our approach allowed flexibility for the analyst, but this strength is outweighed by the multiplied effort to make personalized data subsets and analysis scripts for each separate database.^16,17^

Another important strength from this approach is that this preserves data privacy without having to share or anonymize IPD. Sharing aggregated statistics still allowed pooling of results, which increased statistical power with increased number of samples. This approach would not have worked for small studies of very high dimensional analysis due to low cell counts, unless they first test loss of information with the ECDF method here. Collaborators would have to find a good balance between the dimensionality of data achieved with the percentage of cells with low counts.^7,47^

## Conclusions

Sharing IPD or performing federated data analysis to make large amounts of medical data interoperable and widely accessible to the medical community would quickly advance our pursuit of precision medicine. However, these methods are still underutilized due to data privacy and data security concerns, underdeveloped guidelines, and legal barriers, particularly with clinical trial data. When IPD or FA is not feasible, we present here an approach to partly harmonize the data and share aggregated statistics to avoid data privacy issues, fear of wrongful data use, and divergent interests.

Statistical analysis showed minimal loss of information with this anonymization method. Sharing IPD could have been considered if data reuse and sharing had been adequately taken into consideration before clinical trial data collection, if harmonized guidelines existed, and if there were interchangeable codes of conduct and proper incentives for data generators to share data. Using a common data model and federated data analysis could be a more feasible option, and sometimes more appropriate if data privacy limits sharing IPD.

However, a common data model does not currently exist for clinical trials, and the current decentralized storage of data requires setup and still has security and privacy concerns. Although the approach used in case study 1 of BigData@Heart requires more effort upfront and a strong collaboration, this is a valid method when sharing IPD or performing in federated data analysis is not feasible.

## Data Availability

The data underlying this article cannot be shared publicly due to privacy and ethical restrictions.

## Abbreviations Used

BD4BO: Big Data for Better Outcomes
CI: confidence interval
diffP: differential privacy
ECDFs: empirical cumulative distribution functions
FA: Federated analytics
GDPR: General Data Protection Regulation
HFrEF: heart failure with reduced ejection fraction
IMI: Innovative Medicines Initiative
IPD: individual patient data
OHDSI: Observational Health Data Sciences and Informatics
RCTs: randomized controlled trials
